# Development and validation of a predictive model for end-stage renal disease risk in patients with diabetic nephropathy confirmed by renal biopsy

**DOI:** 10.7717/peerj.8499

**Published:** 2020-02-11

**Authors:** Lulu Sun, Jin Shang, Jing Xiao, Zhanzheng Zhao

**Affiliations:** Nephrology Hospital, the First Affiliated Hospital of Zhengzhou University, Zhengzhou, Henan province, China

**Keywords:** Diabetic nephropathy, Type 2 diabetes mellitus, Risk equation, Risk factors, Chronic kidney disease, End-stage renal disease, Renal biopsy

## Abstract

This study was performed to develop and validate a predictive model for the risk of end-stage renal disease (ESRD) inpatients with diabetic nephropathy (DN) confirmed by renal biopsy. We conducted a retrospective study with 968 patients with T2DM who underwentrenal biopsy for the pathological confirmation of DNat the First Affiliated Hospital of Zhengzhou University from February 2012 to January 2015; the patients were followed until December 2018. The outcome was defined as a fatal or nonfatal ESRD event (peritoneal dialysis or hemodialysis for ESRD, renal transplantation, or death due to chronic renal failure or ESRD). The dataset was randomly split into development (75%) and validation (25%) cohorts. We used stepwise multivariablelogistic regression to identify baseline predictors for model development. The model’s performance in the two cohorts, including discrimination and calibration, was evaluated by the C-statistic and the *P* value of the Hosmer-Lemeshow test. During the 3-year follow-up period, there were 225 outcome events (47.1%) during follow-up. Outcomes occurred in 187 patients (52.2%) in the derivation cohort and 38 patients (31.7%) in the validation cohort. The variables selected in the final multivariable logistic regression after backward selection were pathological grade, Log Urinary Albumin-to-creatinine ratio (Log ACR), cystatin C, estimated glomerular filtration rate (eGFR) and B-type natriuretic peptide (BNP). 4 prediction models were created in a derivation cohort of 478 patients: a clinical model that included cystatin C, eGFR, BNP, Log ACR; a clinical-pathological model and a clinical-medication model, respectively, also contained pathological grade and renin-angiotensin system blocker (RASB) use; and a full model that also contained the pathological grade, RASB use and age. Compared with the clinical model, the clinical-pathological model and the full model had better C statistics (0.865 and 0.866, respectively, vs. 0.864) in the derivation cohort and better C statistics (0.876 and 0.875, respectively, vs. 0.870) in the validation cohort. Among the four models, the clinical-pathological model had the lowest AIC of 332.53 and the best *P* value of 0.909 of the Hosmer-Lemeshow test. We constructed a nomogram which was a simple calculator to predict the risk ratio of progression to ESRD for patients with DN within 3 years. The clinical-pathological model using routinely available clinical measurements was shown to be accurate and validated method for predicting disease progression in patients with DN. The risk model can be used in clinical practice to improve the quality of risk management and early intervention.

## Introduction

Diabetes is a common metabolic disorder around the world (Liu et al., 2019). In China, the overall prevalence of adult diabetes in 2013 was reached approximately 10.9% (Wang et al., 2017). Diabetic nephropathy (DN), which is one of the most important complications of diabetic patients, is found in approximately 35% of type 2 diabetes mellitus (DM) cases and increases mortality (Wanner et al., 2016). In Europe and the United States, diabetic kidney disease (DKD) accounts for about 45% of the patients with end-stage renal disease (ESRD). DKD has become the leading cause of ESRD (Atkins & Zimmet, 2010). The prevalence of DKD in China is also on the rise, and it has become the second cause of end-stage renal disease. As we all know, renal biopsy is the gold standard for diagnosing the pathological patterns. The process from the confirmation of DN by renal biopsy to the progression of renal insufficiency is very short. The median renal survival time was 37.2 months for patients with proteinuria in the nephrotic range and 73.2 months for non-nephrotic range proteinuria (Jiang et al., 2019). Because of its complex metabolic disorder, once it develops into end-stage renal disease, it is often more difficult to treat and control than other kidney diseases, so timely prevention and treatment is of great significance for delaying the progression of diabetic nephropathy. It is imperative to identify patients with DN early and implement targeted management to prevent this progression.

There are many previous studies on the development and validation of risk models that predict the progression of chronic kidney disease (CKD). Renal risk prediction models already exist for patients with advanced CKD (CKD; stages 3–5), such as KFRE model, which was established by Tangri et al. in 2011 (Tangri et al., 2011a). Without considering the etiology of CKD, the model just used four clinical variables (age, gender, glomerular filtration rate and urinary albumin/urinary creatinine ratio) to make predictions. It has been validated in more than 30 countries, but still has not been validated in China; besides, it only targets advanced CKD patients other than DN (Yamanouchi et al., 2018). Another renal risk equation has recently been published for those with type 2 diabetes mellitus (T2DM) based on data from the ADVANCE clinical trial (Jardine et al., 2012), but they do not touch the indicators such as serum creatinine (Scr), pathological parameters or albuminuria.

However, there are some limitations of the existing models. First, the models were predominantly derived from Caucasian populations and the general population (Echouffo-Tcheugui & Kengne, 2012). Their validity in Asians with T2DM remains unclear due to limited data on this population. Second, most of the subjects had T2DM and no clear diagnosis of DN. There is a need to develop a prediction model that can be used to identify patients who need therapy to arrest progression during the time between renal biopsy and the occurrence of ESRD.

We therefore aimed to derive and validate a model to predict the 3-year risk of end-stage renal events, including dialysis, renal transplantation, or death from renal failure, among people with DN without advanced kidney disease within a secondary care context to prevent or slow the progression from DN to ESRD.

## Materials & Methods

### Study population

**Figure 1:**
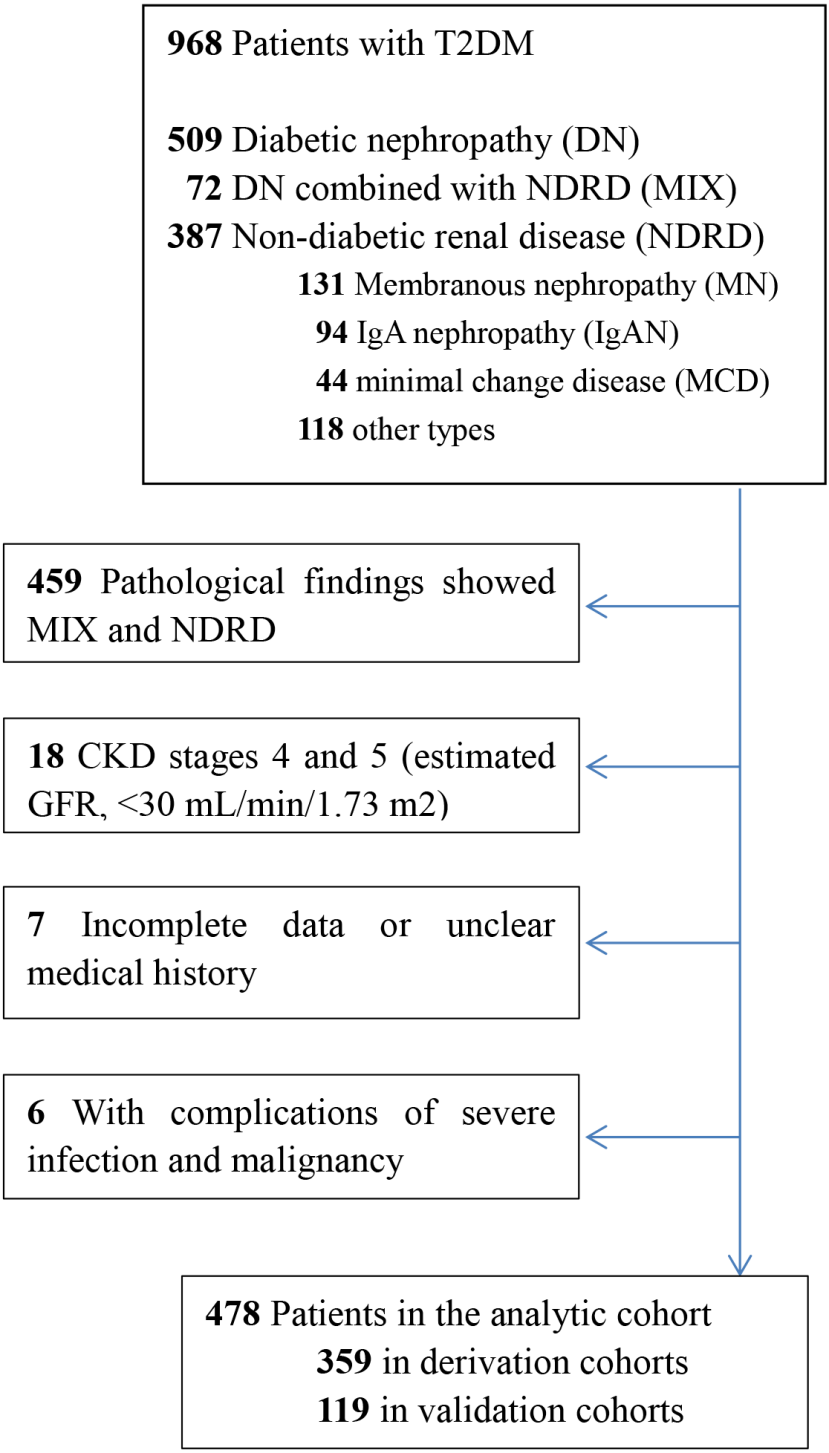
Consort diagram of the 968 patients with type 2 diabetes. Enrollment flowchart of the analytic cohorts used for the derivation and validation of the risk prediction models.

Among the 968 patients with T2DM who underwent renal biopsy between February 2012 and January 2015 at the First Affiliated Hospital of Zhengzhou University, a total of 478 patients were enrolled. Patients were divided into two cohorts: the derivation cohort, which included 75% of the patients and was used to develop the model, and the validation cohort, which included 25% of the patients and was used to test the performance of the model. The inclusion criteria were as follows: age of 18 years or older at the time of renal biopsy (male or female), biopsy-proven renal lesion and T2DM, and pathological findings showing isolated DN. The exclusion criteria were as follows: incomplete data or unclear medical history, with complications such as severe infection and malignancy, pre-existing renal replacement therapy, renal transplantation, or CKD stages 4 and 5 (estimated GFR, <30 mL/min/1.73 m^2^) at the baseline, and pathological findings showed DN combined with NDRD. The enrollment flowchart was showed in Fig. 1.

### Outcomes

The primary outcome was a fatal or nonfatal ESRD event (peritoneal dialysis or hemodialysis for ESRD, renal transplantation, or death due to chronic renal failure [CRF] or ESRD). ESRD was defined as (1) death due to diabetes with renal manifestations or renal failure; (2) hospitalization due to nonfatal renal failure; and (3) an estimated GFR <15 ml min^−1^ 1.73 m^−2^ (National Kidney Foundation, 2002). Outcome events were obtained from patient records during hospitalization and outpatient telephone follow-up until December 2018.

### Data collection

The risk variables involved in building the renal models included the following clinical characteristics: sex; age; medical history of DM and hypertension; laboratory parameters, including pathological grade (Class I, II a, II b, III, and IV represented as 1, 2, 3, 4, and 5 respectively), hemoglobin (Hb) levels, albumin (ALB) levels, hemoglobin A1c (HbA1c) levels, blood urea nitrogen (BUN) levels, serum creatinine (Scr) levels, uric acid (UA) levels, cystatin C (CysC) levels, the estimated glomerular filtration rate (eGFR), 24-h urine protein (24hUTP) levels, point total protein (TCr) levels, the urine albumin/creatinine ratio (UACR), total cholesterol (T-cho) levels, triglyceride (TG) levels, HDL levels, LDL levels, serum lipid (HDL/total cholesterol ratio) levels; and inflammatory indicators such as PCT, ESR and CRP, creatine kinase isoenzyme (CKmb), B-type natriuretic peptide (BNP) and renin-angiotensin system blocker(RASB) use. All of these variables were examined at the time of renal biopsy and were reviewed from electronic medical records. Variables with more than 30% of values missing were not included in the analysis. All other missing data were imputed using the multiple imputation technique with the “mice” package in R version 3.5.1. Type 2 DM was defined according to the World Health Organization criteria (Alberti & Zimmet, 1998). DN was diagnosed according to the presence of glomerular hypertrophy, thickened capillary basement membranes, diffuse mesangial expansion, nodular mesangial sclerosis, mesangiolysis, capillary microaneurysm, hyalinosis of afferent and efferent arterioles, fibrin caps, and capsular drops (Shimizu et al., 2013). The pathologic criteria for diagnosis of DN included glomerular basement membrane thickening, mesangial expansion and diffuse glomerulosclerosis, with or without K-W nodules. Other supportive features were exudative lesions, such as fibrin caps, capsular drops or hyaline thrombi. Biopsies diagnosed as DN were classified based on the consensus published by Research Committee of the Renal Pathology Society in 2010 (Tervaert et al., 2010).

### Statistical analysis

In this study, descriptive statistics including means, medians, and proportions are used to describe the characteristics of the derivation and validation cohorts. A univariable logistic regression model was used to estimate the unadjusted odds ratio for each candidate parameter. Candidate variables with a *p* value <0.1 in the univariate analysis were included in the multivariable model. Excluded predictors were reinserted into the final prediction model to further examine whether they became statistically significant. In the derivation analysis, the clinical model and the clinical-pathological model contained cystatin C, eGFR, BNP, Log ACR at biopsy, and pathological grade. The predictors were selected in the model by multivariable analysis. The clinical-medication model and the full model included the same core predictor variables, but also considered RASB use and age at biopsy. The additional predictors were determined based on the existing literature (Barbour et al., 2019; Dunkler et al., 2015; Elley et al., 2013; Low et al., 2017; Miao et al., 2017). Improvement in model performance due to the addition of new candidate variables in a multivariable logistic regression model was tested with metrics indicating the discrimination, calibration and goodness of fit. Several methods were used to evaluate the performance of the prediction model in the validation dataset. The discrimination and calibration of the model was assessed using receiver operating curves, C-statistics, calibration curves and *P*-values from the Hosmer-Lemeshow test. The goodness of fit of the models was assessed by the Akaike information criterion (AIC). The best model was identified by comprehensively evaluating the performance of each model. Finally, we constructed a nomogram to facilitate the clinical application of this information to predict the risk of progression to ESRD within 3 years in patients with DN. The use of nomogram was as follows: Points are assigned for each variable by drawing a straight line upward from the corresponding value to the “Points” line. Then, sum the points received for each variable, and locate the number on the “Total Points” axis. To speculate the patient’s ESRD rate after 3 years, a straight line must be drawn down to the corresponding “ESRD” probability axis.

We used R version 3.5.1 for all statistical analyses. *P* < 0.05 was considered statistically significant. This study was approved by the Institutional Review Board of the First Affiliated Hospital of Zhengzhou University (2019-KY-017), which was exempted from signing informed consent. This study was conducted and reported in line with the Transparent Reporting of a Multivariate Prediction Model for Individual Prediction Diagnosis (TRIPOD) guidelines.

## Results

During the 3 years of follow-up until December 2018, there were 225 outcome events (47.1%). Outcomes occurred in 187 patients (52.2%) in the derivation cohort and 38 patients (31.7%) in the validation cohort. The patients in the derivation cohort and the validation cohort were similar in terms of age, sex, duration of diabetes, duration of hypertension, Hb levels, HbA1c levels, Log ACR, Tcr, Scr, TG levels, HDL-cholesterol levels, LDL-cholesterol levels, serum lipid levels, eGFR, BNP levels and CKmb at baseline. The patients in the validation cohort had lower CRP levels, ESR levels, 24UTP, Cystatin C and PTH at the baseline than those in the derivation cohort (*p* < 0.05) (Table 1).

### Model derivation & validation

The final variables selected in the final multivariable logistic regression after backward selection were pathological grade, Log Urinary Albumin-to-creatinine ratio (Log ACR), cystatin C, estimated glomerular filtration rate (eGFR) and B-type natriuretic peptide (BNP) (Table 2). The prediction performance details and all supporting data for the clinical, clinical-pathological, clinical-medication and the full model are reported in Table 3, which presents the hazard ratios of five risk models with different combinations of predictive variables, clinical-pathological and the full model performed the best with the highest C-statistic 0.865 and 0.866, respectively, in the derivation cohort and 0.876 and 0.875 in the validation cohort, respectively. But the full model, which include age, pathological grade, cystatin C, eGFR, BNP, Log ACR and RASB use showed the poor *P* value of the Hosmer-Lemeshow test with 0.322. The clinical-medication model, which include cystatin C, eGFR, BNP, Log ACR and RASB use showed the poor *P* value of the Hosmer-Lemeshow test with 0.418. The clinical-pathological model, which included pathological grade, cystatin C, eGFR, BNP and Log ACR, performed well with good discrimination and calibration in the derivation cohort and the validation cohort (Table 3). The AUCs of the clinical-pathological model were 0.865 (95% confidence interval (CI) (0.863–0.867)) in the derivation cohort and 0.876 (95% CI (0.874–0.878)) in the validation cohort (Table 3). The predicted probability was not significantly different from the observed probability of ESRD over 3 years of follow-up according to the Hosmer-Lemeshow test (*p* = 0.909) (Table 3).

## Discussion

**Figure 2:**
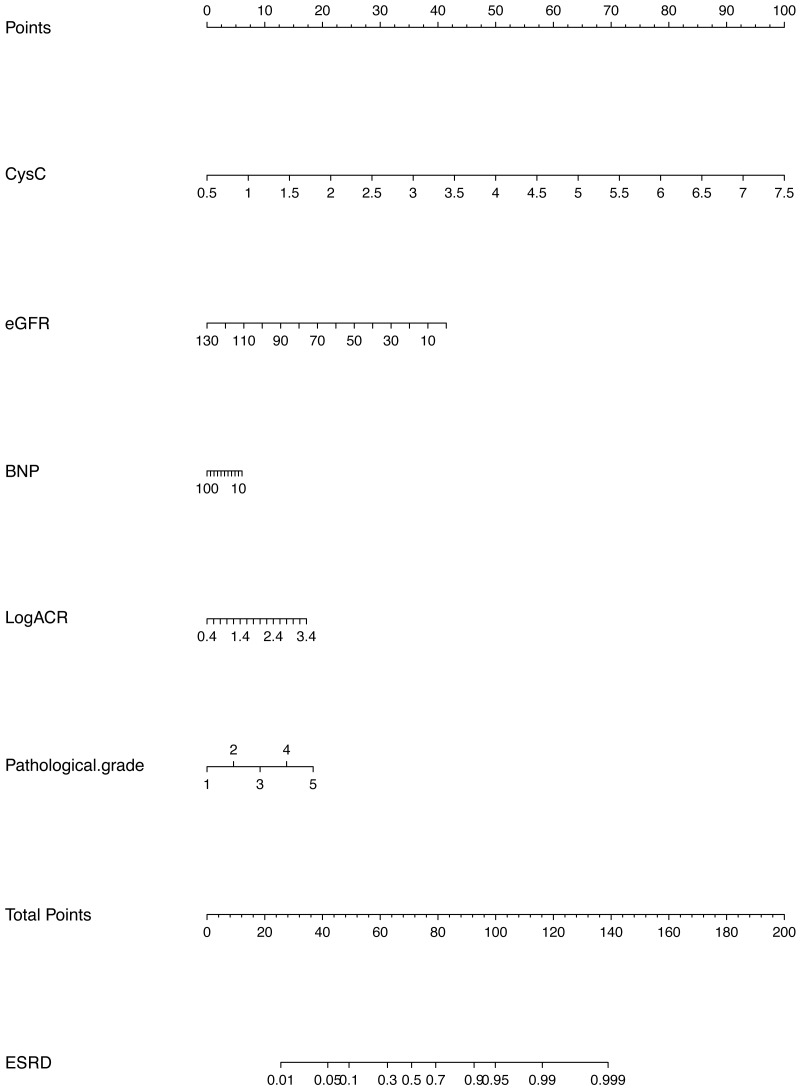
Prognostic nomogram to predict patient’s ESRD rate in 3 years in patients with diabetic nephropathy.

We have developed and validated a prediction model for the risk of ESRD in patients with a pathological diagnosis of DN. The clinical-pathological model showed good performance among the several models. The AUC or C-statistic for clinical-pathological model was 0.865 in the derivation cohort and 0.876 in the validation cohort, showing good discriminatory performance, and the calibration was good, with *p* = 0.909. Our study demonstrated that lower eGFR, higher cystatin C levels, higher BNP, higher Log ACR level and higher pathological grade significantly increased the risk of ESRD in patients with DN. The model incorporating these risk factors can potentially be a simple medical calculator (shown in Fig. 2) for use in clinical practice. Our findings also partially overlap with those from previous studies on prognostic factors in patients with T2DM. For example, in the study of Chinese patients with T2DM and nephropathy in Hong Kong (Keane et al., 2006), the predictors of ESRD included eGFR, uACR and hematocrit (Yang et al., 2006). In the Action in Diabetes and Vascular Disease: Preterax and Diamicron MR Controlled Evaluation (ADVANCE) study, the predictors were sex, diabetic retinopathy, eGFR, uACR, SBP, HbA1c and age at the completion of formal education (Jardine et al., 2012).

Actually, during the long-term clinical practice, DN is usually be given a clinical diagnosis just by the presence of severe edema, macro albuminuria and renal insufficiency in patients with diabetes, so there have been still a lack of studies on pathological changes in DN (Yamanouchi et al., 2018). Even after Tervaert et al. (2010) developed a consensus classification of DN on behalf of the Renal Pathology Society, a limited number of studies on the evaluation of the prognostic value of this classification began to emerge (Mise et al., 2014; Oh et al., 2012; Okada et al., 2012), but no studies have been done to add renal pathology features to the prognostic risk assessment of DN. The importance and necessity of renal biopsy in diabetic patients is still controversial during the practical work (Fiorentino et al., 2017). However, a positive attitude toward renal biopsy is recommended for DM patients. The main clinical significance is probably related to its ability of distinguishing DN from other renal diseases and categorizing the related renal pathology. Indeed, several researches have showed that renal biopsy is meaningful for differentiating pure DN from nondiabetic renal disease (NDRD) because of the different renal outcomes (Chang et al., 2011; Fiorentino et al., 2017; Sharma et al., 2013; Wong et al., 2002).

In our final prediction model, 5 indicators of renal pathological grade, cystatin C, eGFR, BNP and Log ACR were included. In many previous studies (Chen, Wada & Chiang, 2017; Kulasooriya et al., 2018), uACR was generally considered to be a key indicator of the development and prognosis for DN. In our study, it was statistically significant in the univariate analysis and the multivariable logistic regression analysis. The conclusions are consistent with previous studies. In addition, previous studies have shown that BNP was associated with rapid decline of kidney function and incident CKD (Bansal et al., 2015; Mishra et al., 2013). In the study of patients with type 2 diabetes mellitus, showed that elevated NT-proBNP and TnT levels were independently associated with higher risk of ESRD (Desai et al., 2011).

As a classical predictive model, KFRE model is applicable to patients with CKD stage 3–5 (Tangri et al., 2011b), while the model in our study is applicable to patients with diabetic nephropathy diagnosed by renal biopsy pathology. To some extent, the clinical-pathological model in our study is more accurate in population targeting, and its predictive ability is better than KFRE model for patients with type 2 diabetes. However, the KFRE model is a large international study based on multi-center and multi-race, which is more applicable in CKD patients. The model of this study needs more multi-center studies to continuously verify and improve.

**Table 1:** Baseline characteristics of patients at the baseline in the derivation and validation cohorts.

**Variables**	**All** **(*n*=478)**	**Derivation cohort (*n*=359)**	**Validation cohort** **(*n*=119)**	***P***
Age (years)	51 (45, 59)	50 (45.5, 58.5)	51 (45, 59)	0.915
Sex (%)				0.543
Male	292 (61)	216 (60)	76 (64)	
Female	186 (39)	143 (40)	43 (36)	
Duration of DM (years)	9 (3.25, 13)	9 (3, 13.5)	10 (5,13.5)	0.296
Duration of Hypertension (years)	1 (0.1, 4)	1 (0.1, 4)	1 (0.1, 4)	0.518
Hb (g/dL)	104.5 (92, 122)	105 (93, 120)	103 (92, 121.5)	0.892
CRP(mg/l)	1.32 (0.6, 3.38)	1.42 (0.64, 3.49)	1.2 (0.5, 2.41)	0.038
PCT (ng/ml)	0.09 (0.04, 0.2)	0.09 (0.04, 0.2)	0.09 (0.04, 0.2)	0.97
ESR (mm/l)	39 (21, 69)	42 (22, 72.5)	36 (17, 62)	0.047
HbA_1c_ (%) (mmol/mol)	7.41 (6.31, 8.9)	7.49 (6.4, 8.9)	7.4 (6.34, 8.55)	0.393
Alb (g/L)	31.9 (26.85, 37.9)	31.9 (27.25, 37.85)	33.2 (26, 38.45)	0.798
24hUTP (g)	4.21 (2.07, 7.52)	4.3 (2.29, 7.7)	3.45 (1.29, 6.23)	0.041
Log ACR (mg/g)	2.46 (2.01, 2.67)	2.47 (2.03, 2.67)	2.43 (1.9, 2.67)	0.594
TCr (g/g)	4.04 (1.97, 7.2)	4.21 (2.24, 7.27)	4.09 (1.52, 7.07)	0.129
Scr (µmol/l)	123.5 (88, 190.75)	124 (89, 203.5)	123 (82, 189)	0.348
Cystatin C (mg/l)	1.61 (1.24, 2.29)	1.68 (1.24, 2.33)	1.59 (1.21, 2.08)	0.018
TG(mmol/l)	1.7 (1.23, 2.43)	1.65 (1.19, 2.44)	1.7 (1.27, 2.42)	0.982
HDL-cholesterol (mmol/l)	1.12 (0.94, 1.46)	1.13 (0.94, 1.47)	1.12 (0.95, 1.39)	0.744
LDL-cholesterol (mmol/l)	3.25 (2.5, 4.38)	3.25 (2.53, 4.23)	3.13 (2.34, 4.53)	0.694
Serum lipids	0.22 (0.18, 0.29)	0.22 (0.18, 0.29)	0.22 (0.17, 0.3)	0.781
eGFR (ml/min/1.73 m)	49.62 (30.6, 76.76)	48.08 (28.58, 73.56)	53.61 (33.3, 82.66)	0.128
PTH(pg/ml)	50.77 (31.86, 71.56)	55.87 (32.47, 87.87)	39.52 (29, 74.74)	0.018
BNP (pg/ml*10^3^)	1.12 (0.24, 7.61)	1.42 (0.3, 8.87)	1.09 (0.24, 7.23)	0.181
CKmb(ng/ml)	15 (11.4, 23.03)	16.5 (11.1, 25)	15 (12, 23.9)	0.64
Pathological grade (%)				0.55
I	17 (4)	12 (3)	5 (4)	
IIa	34 (7)	25 (7)	9 (8)
IIb	47 (10)	37 (10)	10 (8)
III	303 (63)	222 (62)	81 (68)	
IV	77 (16)	63 (18)	14 (12)	
RASB use (%)				0.786
No	123 (26)	94 (26)	29 (24)	
Yes	355 (74)	265 (74)	90 (76)	
Glucose lowering drugs use (%)				
No	280 (59)	210 (58)	70 (59)	0.564
Yes	198 (41)	149 (42)	49 (41)	
Statins use (%)				
No228 (48)	180 (50)	48 (40)
Yes	250 (52)	179 (50)	71 (60)	
Outcome (%)				0.879
No	253 (52.9)	190 (52.9)	63 (52.9)	
Yes	225 (47.1)	169 (47.1)	56 (47.1)	

**Notes.**

DM Diabetes mellitus Hb Hemoglobin HbA1c Hemoglobin A1c Alb Albumin 24hUTP 24-hour total urine protein ACR Urinary Albumin-to-creatinine ratio Tcr Point total protein TG Triglycerides HDL-cholesterol High-density lipoprotein cholesterol LDL-cholesterol Low-density lipoprotein cholesterol Serum lipids HDL/total cholesterol ratio eGFR Estimated glomerular filtration rate PTH Parathyroid hormone RASB renin-angiotensin system blocker

**Table 2:** Univariable and final multivariable models for ESRD in the derivation cohort.

**Variable**	**Univariable**		**Multivariable**	
	**OR (95% CI)**	***P***	**OR (95% CI)**	***P***
Age (per 15-year increase)	1.41 (0.98–2.03)	0.069		
Sex (male vs female)	0.97 (0.63–1.48)	0.883		
Duration of DM (years)	0.10 (0.97–1.03)	0.987		
Duration of hypertension (years)	1.04 (1.01–1.08)	0.024		
Pathological grade	1.89 (1.45–2.52)	<0.001	1.43 (1.04–1.98)	0.03
RASB use	0.24 (0.14–0.39)	<0.001		
Hb (g/dL)	0.97 (0.96–0.98)	<0.001		
CRP (mg/l) PCT (ng/ml)	0.10 (0.99–1.01) 0.94 (0.70–1.02)	0.666 0.392		
ESR (mm/l)	1.01 (1.00–1.02)	<0.001		
HbA_1c_ (%)	0.83 (0.75–0.93)	0.001		
Alb (g/L)	0.96 (0.93–0.99)	0.003		
24hUTP (g)	1.13 (1.07–1.20)	<0.001		
Log urinary ACR (mg/g)	1.61 (1.12–2.37)	0.012	1.08 (1.01–1.16)	0.04
TCr (g/g)	1.11 (1.05–1.18)	<0.001		
Scr (µmol/l) Urea (mmol/l)	1.02 (1.01–1.03) 1.25 (1.18–1.34)	<0.001 <0.001		
Cystatin C (mg/l)	5.77 (3.94–8.88)	<0.001	4.94 (3.34–7.66)	<0.001
TG (mmol/l)	0.96 (0.86–1.06)	0.430		
HDL-cholesterol (mmol/l)	0.78 (0.50–1.19)	0.256		
LDL-cholesterol (mmol/l)	1.06 (0.93–1.21)	0.390		
Serum lipids	0.24 (0.02–2.12)	0.207		
eGFR (ml/min/1.73 m^2^)	0.95 (0.94–0.96)	<0.001	0.97(0.96–0.98)	<0.001
PTH (pg/ml)	1.01 (1.00–1.02)	<0.001		
BNP (pg/ml*10^3^)	1.08 (1.05–1.12)	<0.001	1.05 (1.01–1.10)	0.01
CKmb (ng/ml)	0.98 (0.97–0.99)	0.04		

**Notes.**

DM Diabetes mellitus Hb Hemoglobin HbA1c Hemoglobin A1c Alb Albumin 24hUTP 24-hour total urine protein ACR Albumin-to-creatinine ratio Tcr Point total protein TG Triglycerides HDL-cholesterol High-density lipoprotein cholesterol LDL-cholesterol Low-density lipoprotein cholesterol Serum lipids HDL/total cholesterol ratio eGFR Estimated glomerular filtration rate PTH Parathyroid hormone RASB renin-angiotensin system blocker

**Table 3:** Performances of the four models with different combinations of predictive variables in the derivation and validation cohorts.

**Variables**	**Clinical model**	**Clinical-pathological model**	**Clinical-medication model**	**Full model**
Age				0.998
Pathological grade		1.270^*^		1.300^*^
Cystatin C	2.728^*^	2.731^*^	2.305^*^	2.663^*^
eGFR	0.970^*^	0.972^*^	0.233	0.973
BNP	1.059^*^	1.057^*^	1.476	1.055^*^
Log urinary ACR (mg/g)	1.077^*^	1.066^*^	1.517^*^	1.066
RASB use			0.841^*^	0.768
**C-statistics**				
Derivation cohort	0.864	0.865	0.842	0.866
Validation cohort	0.870	0.876	0.849	0.875
**Hosmer-Lemeshow test P**	0.558	0.909	0.418	0.623
**AIC**	332.73	332.53	354.44	336.17

**Notes.**

ACR Albumin-to-creatinine ratio eGFR Estimated glomerular filtration rate RASB renin-angiotensin system blocker AIC Akaike information criterion

* statistically significant at *P* < 0.05.

One of the strengths of our study is that the members of the study population were diagnosed with DN, and the diagnosis was confirmed by renal biopsy. In view of the lengthy course of kidney disease in patients with DN, the use of prediction models in both the early and late stages of kidney disease provides a wide window of opportunity for prevention. Our prediction tool can potentially help clinicians identify patients early, before CKD progression starts, thereby enabling them to provide intensive treatment to those at high risk. Second, our study was incorporated a cohort of DN based on pathological changes, this reduced the heterogeneity of the study population to some extent. Our findings will also enrich the currently limited research on the field of pathological information in T2DM (Jardine et al., 2012; Keane et al., 2006; Yang et al., 2006). The third strength is the use of easily available clinical and laboratory information, which are routinely collected in clinical practice. Furthermore, the prediction tool is practical and can be easily applied in the clinical setting, and the presence of a risk prediction model and its integration into clinical practice guidelines have resulted in improved compliance with treatment guidelines and the promotion of personalized treatment.

There are also a few limitations in our study. First, the sample size was only moderate, and the observation period was not long. Second, as with all risk prediction models, it is necessary to conduct multicenter external validation studies before our results can be applied in the clinical setting. Third, our cohort came from the First Affiliated Hospital of Zhengzhou University and hence was not fully representative of the entire population of individuals with DN in China. This limits the generalizability of the findings to other cohorts. Last, the progression of DN to ESRD is a very complicated process, and there may be other risk factors that increase the risk of ESRD that were not explored in this study, such as genetic factors, socioeconomic status, different treatment programs, blood pressure control, glycemic control, diet, exercise and some pathological changes, such as exudative lesions or mesangial lysis, which Furuichi et al. (2018) reported to be strong predictors of ESRD. It is necessary to conduct further and more in-depth research.

## Conclusions

We have developed and validated a model to predict the risk of progression to ESRD in patients diagnosed with biopsy-confirmed DN. The clinical-pathological model demonstrated that pathological grade, cystatin C, eGFR, BNP and Log ACR influenced the disease progression from DN to ESRD. The model performed well and can be a practical and convenient tool for early identification and prognostic prediction among high-risk patients with DN in clinical practice.

##  Supplemental Information

10.7717/peerj.8499/supp-1 Supplemental Information 1 Raw Data

## Additional Information and Declarations

### Data Availability

The following information was supplied regarding data availability: The raw measurements are available as a Supplemental File.
